# A Logical Sequence

**Published:** 1897-05

**Authors:** 


A LOGICAL SEQUENCE.—When the bill to regulate the practice of medicine now before the legislature was under discussion at the Fort Worth meeting of the Texas State Medical Association, April, 1896, in opposing the proposed recognition of homeopaths and eclectics by asking to have them represented on the proposed board of medical examiners, the writer pointed out that it would be but an encouragement to those people to make still further demands; that this recognition was what they ardently desired and were fighting for, and that, if gained, would be but a stepping stone, a paving of the way for still further demands, and that in time we would see them demanding a share of State patronage in the way of holding the medical offices of the great charitable institutions of the State, and *representation on the faculty of the State medical school.*

Our prediction has been verified, sooner than we expected—even before the legislature has disposed of the bill. But—the recognition has been secured; the State Association has conceded the point they contended for,—that they, the "homos" and eclectics are a "school of medicine," and that the great profession of medicine is but *another*, and they are thus emboldened to make the next move.

It came in the shape of an infamous document, circulated anonymously and secretly among the members of the legislature at the most critical time,—pending a discussion of the appropriation for the medical branch of the university. It's animus is perfectly clear, its object apparent. It is an attempt to secure one or more professorships in the medical college by *underbidding*; tacitly, by offering to take such positions for *less money* than is now being paid the professors. In view of the strong tendency towards *retrenchment*, which characterizes the present legislature, these wily fellows thought that "cheap" would be the strongest argument, and they virtually ask to be given places at salaries ranging from $500 to $1000!

As far as can be learned, a Dr. J. S. Downs, whom ever he may be,—said to be president of the State Eclectic Association, a body numbering, perhaps, fifty members, and representing a class of some hundred or more practitioners,—is the author of the document, aided by one Dr. W. E. Ashton, a person whose name we never heard before.*

The document sets forth to the legislature that the State is paying too much for the amount and quality of medical teaching; that professors who get from $2000 to $4000 salary devote only from four to fifteen hours a week to their duties, an average, the document alleges, from $15 to $30 an hour; whereas, (and *here* is the milk in the cocoanut) "good men" can be had to do the same work, or better work, for, respectively, from $500 to $1000 a session!

There is not the shadow of a doubt in our mind that "good men" (?) of *their sort*, could be had for "victuals and clothes;" but like the Indian's estimate of the new minister, it would be "poor pay, poor preach."

The faculty of the college are naturally indignant, outraged. They have felt called upon to notice the thing, and they have published a circular letter in which they say that the document is a tissue of falsehoods from beginning to end. The charges made by the secret enemy are entirely refuted, and at this writing, April 24, we are much gratified to be able to say the legislature *saw through* the transparent thing, and disregarded it utterly. Already they have gone ahead and made the usual liberal appropriation for the maintainence of the medical school. The "bid" was not entertained.

This attempt is but a forerunner of others we may expect. Encouraged by the State Association's recognition of them as *equals*, they, the "homos" and eclectics, will continue their demands till all distinctions are obliterated, and they are taken into the "regular" fold in full fellowship, consultations and all. (How we apples do swim.) The medical profession have no one to blame but themselves. They *paved the way* to this demand, and it is but the logical outcome of the action at Fort Worth, against which we pleaded in vain.

*[Note.—We have since learned that this party failed to pass examination in the Texas Medical College; hence, his venom.]

